# Conceptualising diagnostic liminality: a qualitative exploration of the journey to heart failure diagnosis

**DOI:** 10.3399/BJGP.2025.0698

**Published:** 2026-06-16

**Authors:** Clare R Goyder, Clare J Taylor, Nikki Newhouse, Catherine Pope, FD Richard Hobbs, Lisa Hinton

**Affiliations:** 1 Nuffield Department of Primary Care Health Sciences, University of Oxford, Oxford, UK; 2 Department of Applied Health Sciences, University of Birmingham, Birmingham, UK

**Keywords:** diagnosis, heart failure, primary health care, qualitative research

## Abstract

**Background:**

Heart failure (HF) is a global public health priority. HF diagnosis in primary care is linked to improved outcomes, but most patients are diagnosed in hospital. The pathway to HF diagnosis in primary care is poorly understood.

**Aim:**

To gain deeper understanding of the patient experience of missed opportunities for HF diagnosis and develop recommendations for clinical practice.

**Design and setting:**

A qualitative study with patients recruited through general practice and community nurse clinics in England.

**Method:**

We conducted remote, semi-structured interviews with 24 patients who had a diagnosis of HF. Data were analysed using reflexive thematic analysis.

**Results:**

Three themes were developed: diagnostic liminality and suffering at the threshold of HF diagnosis (when participants were unwell but not yet diagnosed or were unaware of their diagnosis); meaning and framing in the diagnostic moment; and truth-telling and sense-making facilitating the escape from liminality. Although receiving the diagnosis brought relief, it also came as a shock owing to the meanings associated with the term. Some thought HF meant imminent death and was incompatible with living. Participants also described not being properly informed about their diagnosis.

**Conclusion:**

Although life was disrupted by an HF diagnosis, the diagnosis did not enable the transition from liminality. It was truth-telling, in combination with careful explanation, that facilitated the shift from diagnostic liminality. Through sense-making, participants were able to build an understanding of what an HF diagnosis actually meant for them and their future. Clinicians have a vitally important role in guiding patients away from diagnostic liminality through prompt HF diagnosis and thoughtful communication.

## How this fits in

Although billions of pounds have been spent worldwide developing effective drug treatments for heart failure (HF) there has been little investment in strategies to detect HF at early clinical stages, particularly in primary care. This study explores the patient experience of HF diagnosis and missed opportunities for diagnosis. Findings reveal a state of diagnostic liminality, when participants were unwell but not yet diagnosed or not aware of the diagnosis even though this was documented in their medical records. This highlights the crucial role GPs play, not only in detecting HF earlier, but also in communicating the diagnosis clearly and thoughtfully. Doing so can help patients transition from liminality to clarity, improving both clinical outcomes and patient experience.

## Introduction

Heart failure (HF) is a global public health priority, affecting an estimated 64 million people worldwide, and over a million people in the UK;^1–3^ where it is the most common cause of hospital admission for people aged >65 years, with survival rates worse than for those who have cancer.^4,5^ Missed opportunities to diagnose HF in primary care have been characterised from the Clinical Practice Research Datalink (CPRD); 79% of patients have a diagnosis of HF first recorded in hospital despite the fact that 41% of these patients had seen their GP and presented with an HF symptom.^6^

HF diagnosis in primary care is linked to improved outcomes, including significantly lower risk of all-cause mortality compared with those diagnosed in hospital at later clinical stages.^7–9^ People with HF with the worst prognosis were those admitted to hospital with worsening symptoms who were not known to have HF in primary care.^7^ Timely diagnosis of HF in primary care is therefore an urgent clinical priority, highlighted in the NHS Long Term Plan, critical now there is increasing evidence of effective early treatments.^10,11^

Our previous study ‘From Breathless to Failure’ highlighted the tortuous diagnostic journey from symptom onset to diagnosis for patients with HF a decade ago, but HF diagnosis continues to be delayed within primary care.^12^ A recent propensity-matched analysis confirmed that hospital HF diagnosis is associated with substantially increased long-term costs but the human cost of missed opportunities for HF diagnosis is not well understood.^13^ In order to improve the detection of HF in primary care, we need to understand the contemporary patient journey to better develop recommendations for clinical practice. This study addresses these important clinical and research priorities.

## Method

This was a qualitative study comprising 24 semi-structured remote interviews and reported in accordance with Standards for Reporting Qualitative Research.^14^

### Patient and public involvement

Four female and six male members of the Oxford Heart Disease Patient Advisory Group with experience of heart disease informed the study design and contributed to data interpretation.

### Study participants

Adults aged >18 years with a diagnosis of HF in their primary care records were invited to participate. This included patients with the full spectrum of HF diagnoses listed under the Quality and Outcomes Framework HF register, including preclinical HF, such as left ventricular systolic dysfunction. A maximum variation sample was recruited to include participants with HF with reduced ejection fraction (HFrEF) and HF with preserved ejection fraction (HFpEF), participants of different ages and socioeconomic and medical backgrounds, as well as those from diverse ethnicities. Participants included those with an HF diagnosis made within the last year and those diagnosed less recently. Participants with varying HF symptom severity, including all New York Heart Association stages, were also included. Participants needed to be well enough to participate in an interview and patients with dementia were excluded as it was felt that it might be difficult for this patient group to recall details surrounding their HF diagnosis.

### Recruitment

Participants were recruited from primary and secondary care between January 2021 and June 2022. The National Institute for Health and Care Research Clinical Research Network Thames Valley supported primary care recruitment. Four GP practice sites were selected purposively, and included inner-city practices and those in smaller towns that had a more rural catchment. GP practices functioned as patient identification centre (PIC) sites. Secondary care participants were recruited through community Heart Failure Specialist Nurses (HFSNs) based at two hospital sites that also functioned as PIC sites. The PIC sites included formed part of the participant identities used, for example participant A4 was recruited from PIC site A. The HFSN team distributed participant information leaflets to eligible potential participants who they saw either in their own homes or for a hospital appointment. Recruitment continued until the data collected had reached sufficient information power.

### Data collection

The protocol and ethical approvals were adapted to remote methods because of COVID restrictions. Semi-structured interviews were conducted either using Microsoft Teams or by telephone according to the participants’ preference.

At the start of each interview participants were asked to describe their experience of HF diagnosis in as much detail as they could. This was then followed by more structured questions using a topic guide developed and refined through consultation with the patient and public involvement group. Interviews were audio-recorded, anonymised, and transcribed verbatim. Interviews were carried out by the project lead, a female researcher and GP who had training and experience in qualitative research. A reflexive diary was kept throughout the study, which included reflections on the dual role of being a researcher and a GP.

### Data analysis

Data were analysed using reflexive thematic analysis, an interpretative process, that included data immersion, annotating the transcripts, progressing to coding, re-coding, reflection, and theme development.^15^ To illustrate this with an example, one of the initial codes was ‘distress’ and this was allocated to the quote *‘when the GP told me that I was on the optimum medication for my COPD* [chronic obstructive pulmonary disease] *and there was nothing more they could do’.* When all the quotes coded as ‘distress’ were re-examined together, we discovered that patient-reported distress was associated with the experience of being ‘in-between’ different diagnostic states. We focused our thinking on this ‘gap’ and on the lived experience of being on the threshold of diagnosis and this led to the development of the theme of ‘diagnostic liminality’.^15^ Three coauthors read transcripts independently. Analysis and theme development were collaborative involving discussions with the coauthors. NVivo (version 12) qualitative data-management software was used to facilitate data analysis. Regular team meetings were used to scrutinise coding and theme development, and team members were asked to question each other’s assumptions and particularly to consider how the lead author’s positionality was shaping the analysis. This, in conjunction with the lead author’s reflexive diary, was used throughout the analytical process to explore bias and underpinning assumptions.

We moved from inductive coding of the data to a more deductive process (and we acknowledge that this can be a continuum).^15^ We integrated existing theory to help explain the data and identify and further develop themes. Data analysis was therefore informed by theories of liminality, biographical disruption, and the sociology of diagnosis.^16–20^ Liminality is an anthropological concept denoting a ‘state of in-between-ness’, with the word ‘limen’ originating from the Latin for ‘threshold’.^16,21^ Augé describes non-places or liminal places such as airport lounges that are transient spaces that can ‘dislocate identity’*.* ^17^ The medical sociologist Bury conceptualised chronic illness as a particular type of *‘*disruptive event*’* that triggered a fundamental rethinking of people’s self-concept or personal biography.^20^ Jutel has argued for the development of the sociology of diagnosis beyond existing frameworks (such as medicalisation), emphasising that diagnosis is a powerful social tool that deserves its own sociological analysis.

## Results

In total, 24 participants were recruited: 19 through four GP practices in the Thames Valley region; five participants were recruited through HFSNs in Oxfordshire (Table 1). Interviews lasted approximately 45–60 min.

**Table 1.** Characteristics of participants (*N* = 24)

Characteristic	Participants, *N* (%)
**Sex**	
Female	11 (46)
Male	13 (54)
**Age group (years**)	
50–59	1 (4)
60–69	6 (25)
70–79	11 (46)
80–89	5 (21)
90–99	1 (4)
**Ethnicity^a^**	
White British	21 (88)
Black and minority ethnic	2 (8)
Non British	1 (4)
**Socioeconomic status^b^**	
1 (Most deprived)	0 (0)
2	3 (13)
3	0 (0)
4	1 (4)
5	2 (8)
6	3 (13)
7	2 (8)
8	5 (21)
9	3 (13)
10	5 (21)
**Time since HF diagnosis (years**)	
<1	3 (13)
>1–5	15 (63)
>5–10	3 (13)
>10	3 (13)

a Ethnicity was self-identified. ^b^Calculated using Index of Multiple Deprivation decile based on participants’ postcode. HF = heart failure.

Three main themes were developed from the data and are presented:

- diagnostic liminality and suffering at the threshold of diagnosis;
- meaning and framing in the diagnostic moment; and
- truth-telling and sense-making in the escape from liminality.

### Theme 1: Diagnostic liminality and suffering at the threshold of diagnosis

Missed opportunities for diagnosis, or not being aware of an HF diagnosis, resulted in a liminal existence. These situations were conceptualised as diagnostic liminality (the state of ‘in-between-ness’) that participants described as oscillating between different states.^21^ On the threshold of diagnosis, they were caught in an ambiguous place. Unwell, but not diagnosed, or unaware of the diagnosis, they were unable to live or function normally and without an explanation for why.

Common symptoms of HF often developed insidiously until they became more severe and there was a realisation that something was wrong, for example, a shortness of breath when walking up an incline but not on the flat. For those with more severe HF, symptoms worsened until everyday activities became a physical struggle. Difficulty breathing when lying flat caused sleep disruption. It became too difficult to breathe and walk simultaneously, so walking was avoided, and it became easier to only travel by car or avoid going out at all.

Sometimes there was a specific moment where the situation reached a tipping point that initiated help-seeking strategies. For most participants, this meant contacting their GP. Although HF was recognised quickly by some GPs, this was not always the case, resulting in missed opportunities for diagnosis. For participant E21, her breathlessness and fatigue were attributed to COPD secondary to smoking. She gave a harrowing account of this time:

*‘I battled through those years* […] *I went back to the GP’s quite often saying that it’s getting worse and I remember basically when the GP told me that I was on the optimum medication for my COPD and there was nothing more that they could do* […] *this was now just the last kicks of a dying horse.’* (E21)

Eventually, her hairdresser and her pharmacist persuaded her to attend the emergency department (ED) and this led to her finally getting an HF diagnosis. She had been so unwell that she was certain she was dying. She felt that if she:

*‘*[…]*couldn’t breathe any more, you know, I can’t be alive.’* (E21)

Lay advocacy was also echoed by other participants who described how their help-seeking was prompted by family intervention. Participant A4 had recently completed chemotherapy and radiotherapy for breast cancer and then found that she was too breathless to sleep and finally to even speak. Despite the 111 phone line advising that she should be seen urgently, her GP could not offer her an appointment for 5 days:

*‘And so whilst my partner was telling me that he felt I needed to go to ED, I felt I was perhaps being unreasonable and that I wasn’t that ill and then I spoke to my sister-in-law and she heard in my voice and she just said, “No, go to ED now.’’* (A4)

Diagnostic liminality was associated with psychological and physical suffering. Being unwell without validation or explanation took its toll. Participants questioned themselves, wondering if they were imagining symptoms; indeed, participant A4 recalls being told by an ED doctor that she did not ‘look like she had HF’ and being initially discharged and diagnosed with a chest infection. One participant who did not speak English as a first language was very frustrated as he felt that no effort was made to explain the diagnosis to him. For another participant, there was a suggestion that this lack of a clear explanation for symptoms contributed to a depressive episode:

*‘I wish someone had sat down with me and explained it all but I think I went into a, like a depression after that* […] *you know, if I had somebody* […] *sat with me and explained it all maybe I’d have felt a lot better.’* (E20)

### Theme 2: Meaning and framing in the diagnostic moment

Our data revealed contradictory responses to diagnosis: relief and despair. Although having an explanation for symptoms brought relief, there was shock and despair in response to hearing the term ‘heart failure’ because this was associated with imminent death. The use of language, including euphemistic terms and how an HF diagnosis is framed in the diagnostic moment, had the potential to cause additional distress and confusion, further deepening the liminal experience.

Participants described relief and reassurance in finally knowing what was wrong, especially after seeking this for so long. An accurate diagnosis triggered the initiation of appropriate treatment. Treatment then brought physical relief, the easing of a heavy symptom burden, and an end to the distress and fear associated with this:

*‘Oh, it was absolutely, it was just such a relief because, you know, even I, I still think calling it heart failure is fairly scary but actually just knowing what was wrong with me, and being able to* […] *walk and breathe and talk all at the same time. It was fab.’* (A4)

There was relief in the validation that something was indeed wrong, although this was mixed with despair, further deepening a liminal existence. Drivers of this despair included the meanings associated with the term ‘heart failure’, which for many was utterly frightening, shocking, and associated with death. For participant B6 this was a ‘death sentence’.

Participants’ associations between the term ‘heart failure’ and death/dying are exemplified in the following quotes:

*‘Well, I suppose being a layman in these matters, if you hear you’ve got heart failure, it’s, it’s, it almost sounds as if you’ve got a death sentence. I don’t feel that now. Yeah, yeah, I thought, “Oh this is, I’m on the way out”, I thought.’* (B6)

*‘Oh god, I’m going to die.’* (A2)

*‘I thought, “For goodness sake, I’m, I’m on the way out.”’* (B7)

*‘Is it not working, you know and so it’s not working at all shouldn’t I be dead?’* (D17)

*‘A death sentence sort of thing.’* (E16)

*‘That eventually it’s going to give up.’* (E15)

*‘Something that fails is broken, and if your heart is broken, you know, it sounds like they’re telling you you’re on your way out.’* (E21)

*‘The end, like a full stop to me.’* (E16)

*‘Not gonna last that long.’* (E13)

Participants reported that clinicians sometimes avoided using the term ‘heart failure’ in the diagnostic moment. There was a tendency to use euphemistic language or alternative medical terms instead such as ‘water retention’ (A24). With worsening HF, this language escalated so participants became *‘full of water’* (B5) or even *‘submerged in it’* (E21). A participant stated:

*‘I was told about this ejection fraction 13 years ago this month, 13 years, that was basically very mega heart failure, but it was never, never put in those terms. It was just one of the many conversations I had with many doctors and consultants.’* (A2)

Despair and diagnostic liminality deepened further when participants found out that they had HF indirectly, as exemplified in the cases of two participants (E13 and E16).

Participant E13 described receiving a medical letter written from a hospital doctor to a GP and copied to the patient for information. One-way, indirect communication removed the option to ask questions or further understand what was wrong:

*‘I had no idea that I had heart failure until he sent me that letter so it wasn’t face to face* […] *He didn’t really tell me that I had heart failure. Wasn’t* [it] *a bit of a crass way of telling you that you had heart failure, not, not any other explanation for it, you know.’* (E13)

For participant E16, it was 2 years between discovering she had an ‘abnormal echo’ and understanding that she actually had HF, despite asking her GP what this meant and whether she needed any follow-up. While having an endoscopy she described how:

*‘When I was in for the procedure the doctor said he couldn’t give me any* […] *not very much* [… of the] *drugs to relax me because he said of your heart failure* […] *And that was the first time I’d heard heart failure.’* (E16)

Specific difficulties arose when there was a documented diagnosis of HF in the medical record, but participants were unaware of this. This participant saw a GP he had not previously met and who revealed his diagnosis to him:

*‘Whilst consulting for another issue,* [the] *GP said, “You’re doing as well as can be expected for someone who has heart failure”, and no one had ever told me that before.’* (A2)

Many participants with HF still viewed themselves as healthy; however, the language and framing in the diagnostic moment often contrasted with these perceptions. One participant, a retired physical education teacher, echoed the sense of loss that this diagnosis can bring:

*‘I was told well I’ve got to accept what condition I’m in because you’ll be, you’ll soon be 70 and “you’ll be quite happy sitting in the chair for the rest of your life”. Well that was a bit of a shock to me because I didn’t really wanna sit, be in a situation where I would sit in the chair all the time. I like to know I can do things.’* (A1)

For the rest of his interview, this participant referred frequently to this instruction as being synonymous with having HF, explaining that he had a condition that meant he would need to *‘sit in a chair’* (A1) for the rest of his life. For him, this future was embodied within the diagnosis of HF.

### Theme 3: Truth-telling, sense-making, and understanding in the escape from liminality

This final theme explores how truth-telling and explanation facilitated sense-making, learning, and understanding. All these components were required for participants to escape from diagnostic liminality.

Participants who had previously been unaware of their diagnosis, or for whom it had been obscured behind euphemisms, described how they were left with a sense of being *‘fobbed off’* (E16) thus prolonging the liminal existence. They advocated strongly for the importance of truth-telling, arguing that clinicians should use the term ‘heart failure’, especially when this is included in their medical records:

*‘Straightforward and honest*[communication was what I wanted to hear]. *Not telling me a ‘heart murmur’, ‘abnormal’, all these words and not once* [actually saying ‘heart failure’] *Some people might not wanna know* [... but I think, being told] *straight out the truth, it would be, you know better.’* (E16)

*‘In the end, I mean I, you know, my initial reaction was, “Oh that’s a bit brutal”, but of course I mean yeah I mean somebody should have told me years ago.’* (A2)

Different approaches to communication and information-sharing, including discussions with HFSNs, observing heart function during echocardiography, and educational resources, such as patient information leaflets, facilitated further sense-making. Participants described how this communication and information helped to *‘take the poison out of it’* (A2) and, through this learning, they realised that HF was not a death sentence. A key component of this was that participants were given the opportunity to ask questions.

Eventually, over time, most participants described gaining a better understanding of HF and its implications. There was a collective understanding that a better description for HF is the heart is not pumping as well as it should, but that it was still functioning; and there was reassurance in the realisation that it is not a complete shutdown:

*‘And now, I think of it being, that it simply means that the heart is failing to operate as well as it should. And that’s all, that’s all I take on from it now, it’s not operating as well as it should.’* (D11)

Slowly, as their understanding increased, participants described being more comfortable with the term ‘heart failure’. Their language evolved so words such as *‘sub-optimal’* (A2) or an ‘*inefficient heart’* (A2) were used in preference, and one participant suggested that it was more accurate to say that he had *‘a degree of heart failure’* (B6). A participant stated:

*‘I, I think if you explain what heart failure was they’d probably, they wouldn’t be frightened because of explaining like this being, you know parts of the heart aren’t working as they should be working or have to work a bit harder because of this. I think that, that would reassure them a little bit.’* (E18)

Overcoming diagnostic liminality through improved communication and understanding allowed for helpful discussions about prognosis, facilitating a transition towards acceptance for many. In time, patients with HF may face other liminal phases such as the transition towards end of life; however, the focus for most participants, once they had overcome diagnostic liminality, was on the present:

*‘It’s precious that, that’s what I find, I wanna stay alive as long as I can and if I could do anything to prevent an earlier death I would try to, try to be healthier and well just live a better life I suppose.’* (E16)

## Discussion

### Summary

This study provides critical insights into the patient experience of HF diagnosis. Participants described a liminal existence at the threshold of diagnosis, marked by physical and psychological suffering. Oscillating between different states, they were unwell but not diagnosed, or unwell but not aware of their diagnosis, alive but not able to live or function normally, and searching for an explanation for why this was. Together these situations are conceptualised as diagnostic liminality.

Clinical deterioration triggered intervention from social or family networks, escalating help-seeking strategies, finally leading to HF diagnosis. Although receiving the diagnosis brought some relief, for many it came as a shock owing to the meanings associated with the term ‘heart failure’. For almost all participants, HF initially was interpreted as meaning imminent death or dying. Poor communication at the time of diagnosis further deepened the liminal state. Truth-telling combined with careful explanation and information resources facilitated an escape from diagnostic liminality and, for some, there was then a transition from liminality to acceptance. Through sense-making, participants developed an understanding of what HF diagnosis actually meant for them and their future. Our study highlights the crucial role clinicians can play in guiding patients out of diagnostic liminality through prompt HF diagnosis and clear and thoughtful communication.

### Strengths and limitations

This study makes a significant contribution to research in this field. Although quantitative methods have identified missed opportunities for diagnosis, findings from this study increase our understanding of the impact of these. This study develops the concept of diagnostic liminality — as a state of ‘in-between-ness’ — a lens through which to understand the patient experience of diagnosis; and this provides a useful focus for future analysis. Most importantly, this study highlights the suffering associated with diagnostic liminality and offers important insight into why thoughtful communication of diagnosis matters. Moreover, from this evidence, directly applicable recommendations for clinical practice have been developed, to improve the future experience of HF diagnosis.

Further strengths include the recruitment of a maximum variation sample from primary and secondary care in both urban and more rural areas, although only two participants from black and minority ethnic communities were included. Efforts were made to increase socioeconomic and ethnic diversity with purposive sampling, but participants from the lowest deprivation deciles were not included because no participants from this group volunteered to be interviewed. Recruitment strategies should ensure this group are represented in the future. A wider geographical area for recruitment might have improved this as this study was limited to the Thames Valley region; this was pragmatically driven as initially all interviews were planned to be face to face and therefore affected by travel and financial limitations, but remote methods introduced during the COVID pandemic could have broadened geographical spread. We did not specifically recruit any participants with HF with mildly reduced ejection fraction and we did not go into detail to compare experiences of those with HFpEF versus HFrEF. We also restricted recruitment to those participants included on GP HF registers. This meant that we excluded those people who are not included in HF registers because of coding errors, but they would have offered a unique perspective that was missing from this study.

We also did not specifically explore differences between how patient experience of diagnosis differed with different healthcare professionals, although participants did describe how discussions with HFSNs were helpful and facilitated sense-making. We did not include the perspective of carers in this study, although carers have been the focus of one of our previous studies and offer really vital insights into the diagnostic process, and this study could have been strengthened by including them and triangulating data to inform findings and discussion.^22^

It is important to be mindful of recall bias. Participants were asked to recount details of an experience, which for some had happened years previously. In an attempt to mitigate for this, participants with both a recent diagnosis (<1 year) as well as those for whom the diagnosis was more distant were included and the majority of participants were <5 years post-diagnosis. We observed that many participants for whom years had elapsed since diagnosis could recount in specific detail events and words that were said at the time, reflecting the significance of the event in the context of the participants’ lives. In general, participants who were >5 years post-diagnosis tended to report more challenging experiences than those diagnosed more recently, although this observation was not examined in detail. The study does not include clinicians’ perspectives so descriptions of these conversations do not include the intent of the clinicians involved. Some participants were diagnosed during the COVID pandemic and this will have had an impact on their experiences, particularly as clinical communication during this time was particularly challenging.

### Comparison with existing literature

Missed opportunities for HF diagnosis have been characterised quantitatively using routinely collected general practice data.^6^ A large CPRD study demonstrated that median times from presentation with HF symptoms to referral exceeded those recommended by the National Institute for Health and Care Excellence (NICE) (2–6 weeks), by over 6 months for most patients.^23^ Furthermore, in the Diagnose-NP study we found the median time between any natriuretic peptide test and HF diagnosis was 101 days, far exceeding the recommended timelines in the NICE guidelines.^24^ This delay is consistent with the data in this study; moreover, there is evidence of a ‘non-linear’ pathway to diagnosis, including HF also being missed in the ED.^25^ These findings have important clinical implications because compliance with NICE guidelines, such as prompt HF recognition, referral, and initiation of medications, correlates with improved outcomes for patients.^25,26^

A cross-sectional survey by Tayler and Ogden found that GPs preferred to use euphemisms rather than the term ‘heart failure’ and concluded that *‘the choice of language may present a dilemma for doctors’* because they are torn between being open and being protective of their patients.^27^

This study sits alongside a body of work in the sociology of health and illness on diagnosis and biographical disruption (Box 1). Additionally, qualitative studies have provided important insights into the diagnostic journey to HF, including the normalisation of initial symptoms by participants causing delayed help-seeking and the important role of informal carers to escalate help-seeking strategies.^12,22^ There are correlations with our findings regarding the diagnostic meaning of HF and the variable understanding of, and challenges associated with, the term ‘heart failure.’^12,28^

Box 1. **Comparison with existing theory**

- Sociological approaches to diagnosis and the role it plays in health care and society have much to contribute.^19,38,39^ Balint observed the distress experienced on being told there is ‘nothing wrong’ to a patient’s *‘most burning demand for a name for his illness’*.^40^ Jutel highlights how naming a diagnosis validates and legitimises illness; facilitates access to the sick role and underscores the power of language in the diagnostic moment: *‘The naming of a serious diagnosis is a powerful thing. It changes how we think about our bodies, our disorders, our futures, and even our identities. The naming of a disease can sometimes be more powerful than the disease itself.’* ^36^
- This aligns with Schofield’s observation that a doctor’s words can have a ‘double force’; indeed the language used by clinicians could be recalled years after, particularly for participant A1 who described being told that his heart failure (HF) diagnosis meant that he would need to *‘sit in a chair all day’*.^36,37^ The language and meaning of this further added to his sense of despair and disrupted his sense of identity as a previously very active person. This illustrates the power of a new diagnosis to cause biographical disruption. This was conceptualised by Bury, who observed how chronic illness is a type of ‘disruptive event’ that triggered a fundamental rethinking of people’s self-concept or personal biography.^20^ This also aligns with Fleischman’s observations on the symbolic power of diagnostic language: *‘The verbal act of presenting a patient with a diagnosis is never a simple act of conveying value-neutral biomedical information. It is an act fraught with symbolism, as is the act of referring a patient to a specialist. If a person is told “you have cancer” (or any life-threatening disease) these words irrevocably alter that person’s consciousness, view of the future, relationship with family and friends, and so on.’* ^41^
- Van Gennep’s three stages of rites of passage offer a conceptual framework that has also been used to study the lived experience of multiple sclerosis diagnosis on an individual’s sense of self.^16,42^ Strickland describes how the ‘road to diagnosis’ echoes the pre-liminal self and that the diagnostic moment triggers the transition into the liminal self with the final stage being the post-liminal self.^42^ This sense of a division, marked by the utterance of a diagnosis, was also observed by Fleischman: *‘The utterance marks a boundary. It serves to divide a life into “before” and “after”, and this division is henceforth superimposed onto every rewrite of the individual's life story.’* ^41^
- There is this same division in the data from this study; there is an indelible boundary between before and after diagnosis.^19,41^ However, there is a clear difference between our analysis and that of Fleischman and Strickland because they credit the diagnosis with the ‘power to transform’ and move people on to that different state, which is not evident in the HF data presented here.^41,42^ Bury also observed that in the face of this disruption many will try to overcome it and even to normalise it, and that this is a way to restore meaning.^20,43^ In our data, for many participants, life was disrupted by the moment of HF diagnosis but the diagnosis did not facilitate a transition into the after division; in fact it deepened the liminal state and they remained suspended as ‘threshold people’.^21^ It was only when a diagnosis was explained and understood did participants describe being able to transition away from diagnostic liminality towards acceptance.

### Implications for research and practice

Recommendations for clinical practice are outlined (Box 2). Most importantly, despite having a worse prognosis than many common cancers, HF is not viewed as a malignant condition in the same way, and a shift is needed to challenge this perception that may underpin both diagnostic delay and poor communication at the time of diagnosis.^5,29^

Box 2. **Clinical recommendations**

- The diagnosis of heart failure (HF) must be considered in all patients who present to primary care with symptoms such as breathlessness, ankle swelling, and fatigue.

- It is especially important to consider HF in those who are at increased risk owing to chronic diseases such as diabetes or ischaemic heart disease. Participants in this study who developed HF secondary to previous myocardial infarction were frustrated that they were at increased risk of HF, but the diagnosis was still not considered when they consulted their GP with HF symptoms.
- Diagnostic overshadowing means that HF is often missed in people who also have chronic obstructive pulmonary disease (COPD) as their breathlessness is thought to be because of COPD. Always consider whether people with COPD may have both conditions. Participants in our study had also developed HF after chemotherapy and this is also an important at-risk group to be alert to.
- Robust systems for arranging and following up results of raised natriuretic peptide test results and community echocardiograms are essential. Participant E21 was advised that she required an echocardiogram but this was never organised and she deteriorated in the intervening time.
- Reflecting generally on patient–clinician communication overall and specifically during history taking, one participant suggested clinicians needed to *‘Listen more … and maybe ask more questions. Because I think if the GP had asked me a lot more questions from the start, you know, a lot more would have come out’* (E21).
- Participants described the need for clinicians to thoughtfully and directly communicate the diagnosis of HF to patients and their carers and that, wherever possible, the diagnosis be discussed in a face-to-face consultation to avoid one-way communication strategies. Participants advised that it was important to avoid euphemisms or using medical terminology alone without explanation.
- Participants advised that it is vitally important that careful explanation and a chance to ask questions follows the diagnosis so that they could build an understanding of what HF is and crucially what it means for them and their future.
- One participant finally received her HF diagnosis from a cardiologist when she was admitted to hospital acutely breathless, having had the diagnosis missed twice in the emergency department. She noted the importance of eye contact as part of non-verbal communication skills: *‘He just looked at you straight in the eyes all the time and looked as though he really, really cared about you … somebody who actually was, you know, really interested in what was wrong with you’* (A4).

With the rapid evolution of treatment options for HF, particularly at early clinical stages, the imperative to proactively identify affected patients in primary care has never been more critical, especially for those in underserved communities and in people with COPD who are particularly at risk of hospital HF diagnosis at later clinical stages. HF and COPD frequently coexist (20–70%), with comorbidity predicting mortality. Both cause breathlessness, complicating diagnosis.^30^ Proactive HF detection in COPD is limited.^17,31^ Deeper understanding of the barriers to timely diagnosis, particularly for patients with COPD, is therefore a research priority. Future qualitative research should focus on clinicians and understanding missed opportunities for HF diagnosis from their perspective. Repeating the national survey and focus group study performed in 2003 and 2014 would allow further exploration of the contemporary diagnostic challenges for primary care clinicians including the impact of the Additional Roles Reimbursement Scheme.^32,33^

Furthermore, understanding inequalities in primary care HF diagnosis through analysis of electronic health records is a vital area for future research and is now under way.^34^ HF detection should be more proactive, rather than relying on symptom presentation, and symptom questionnaires have been shown to be an effective case-finding strategy. It may also be beneficial to ask about HF symptoms opportunistically during routine appointments or during annual chronic disease reviews for high-risk conditions.^35^

This study highlights the importance of individualised communication at the time of diagnosis. Using the analogy of liminal places, doctors need to guide patients out of the airport lounge and fly them home — being ‘told’ the diagnosis will not release the patient from diagnostic liminality. Saunders, in the Foreword to Jutel’s book *Diagnosis: Truth and Tales*, advocates that a doctor must: *‘Give that story back to her patient and to do it in a way that allows him to try it on and, if it seems to fit, to incorporate this new version of his story back into the bigger story of his life.’* ^36^ Maintaining thoughtful communication through the digital transformation is essential, particularly as interactions increasingly occur through one-way text messaging and direct patient access to online medical records. This indirect communication does not allow the doctor to give the ‘story back*’* to their patient. However, detailed, careful explanation, with opportunity for discussion, supports sense-making and human connection. This facilitates the transition from diagnostic liminality and as Saunders concludes in the Foreword: *‘It is one of the most important parts of our job. Done well, the patient has a sense of what is happening to them, what is going to happen to them, and what can be done.’* ^36^

In conclusion, by characterising the patient experience of missed opportunities to make and explain HF diagnosis, this study has developed the concept of diagnostic liminality. This concept is helpful for current and future clinicians, researchers, and policymakers alike. It is hoped that this understanding will translate into improvements in diagnostic pathways and better communication between clinicians and patients. Although making an accurate and timely HF diagnosis is very important, this study has demonstrated that being told the diagnosis is not enough. In addition to being ‘told’ of an HF diagnosis, patients need to build an understanding of what this means for them and their future. This is only possible if the diagnosis is carefully explained, and appropriately ‘given back’.^36^ The terminology of HF needs careful consideration and clinicians need to be aware of the meanings patients associate with HF and the heightened impact of words used.^37^ Language used by clinicians in the diagnostic moment matters and can resonate for years, if not for the rest of a patient’s life . This study highlights the crucial role GPs play, not only in detecting HF earlier, but also in communicating the diagnosis clearly and thoughtfully. Doing so can help patients transition from liminality to clarity, improving both clinical outcomes and patient experience.
